# Supplementary material for: An Angiogenic Gene Signature for Prediction of the Prognosis and Therapeutic Responses of Hepatocellular Carcinoma
Source: Int J Mol Sci. 2023 Feb 7;24(4):3324. doi: 10.3390/ijms24043324 (PMC9965274; doi:10.3390/ijms24043324)
Supplement: Supplementary file 1 [file ijms-24-03324-s001.zip › ijms-2160053-supplementary.pdf]

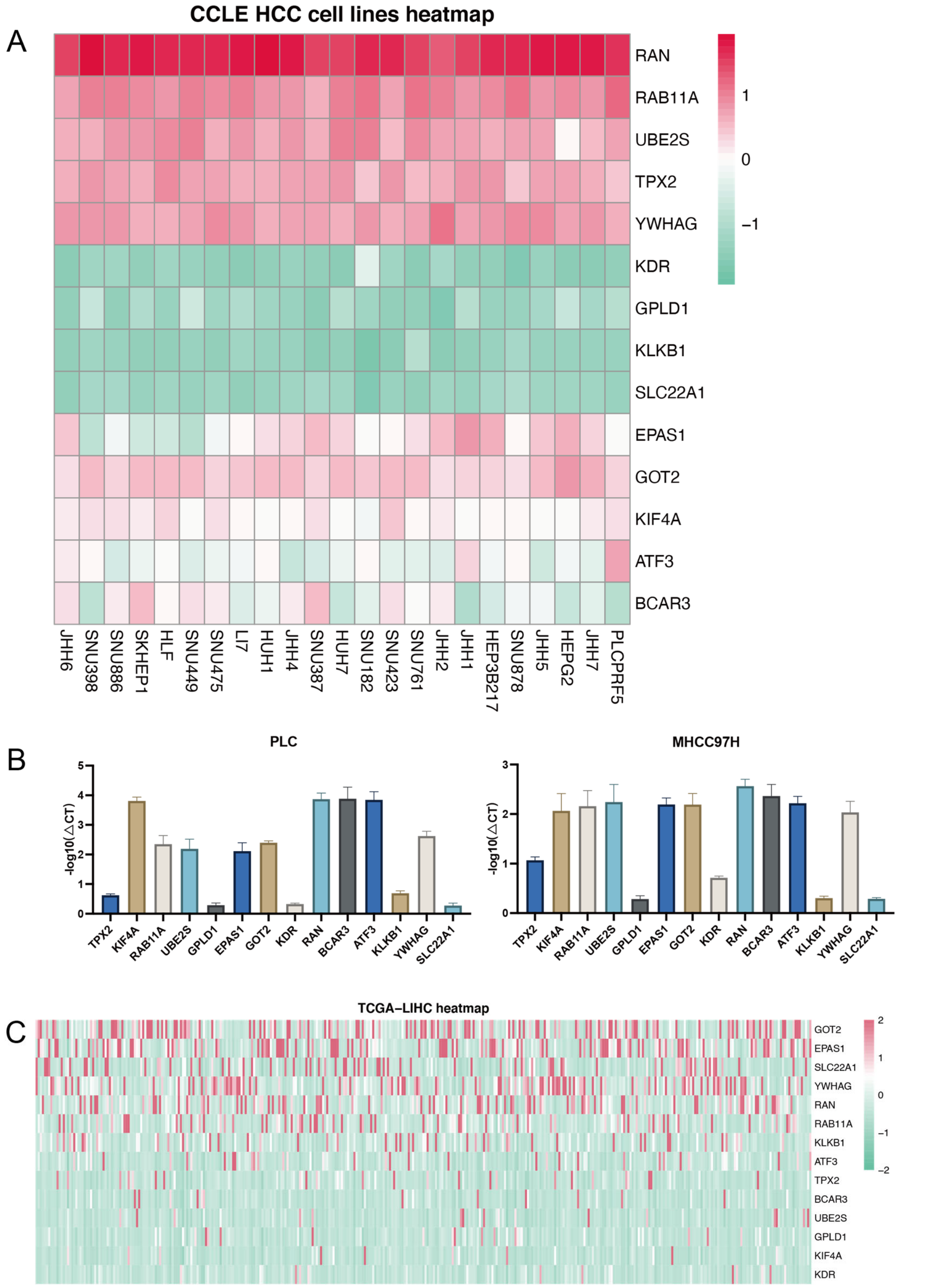

Supplementary Figure S1. The expression levels of the ARPG genes in the cell lines and tumors. (**A**) 14 ARPGs distribution in HCC cell lines from CCLE database. (**B**) Quantitative PCR Shows the expression of 14 ARPGs in RNA samples from two HCC cell lines. (**C**) 14 ARPGs distribution in HCC cohort from TCGA database.
